# The Potential Role of Organ-to-Liver Ratios in Immune-Related FDG PET Imaging. Comment on Panikar et al. Exploratory FDG PET/CT Imaging in mCRPC Patients Treated with Sipuleucel-T ± IL-7: A Phase II Trial Subanalysis. *Int. J. Mol. Sci.* 2026, *27*, 5129

**DOI:** 10.3390/ijms27188178

**Published:** 2026-09-14

**Authors:** Alev Çınar

**Affiliations:** Department of Nuclear Medicine, Ankara Training and Research Hospital, 06230 Ankara, Türkiye; alevcnr@gmail.com

We read with great interest the article by Panikar et al. entitled “Exploratory FDG PET/CT Imaging in mCRPC Patients Treated with Sipuleucel-T ± IL-7: A Phase II Trial Subanalysis” recently published in the *International Journal of Molecular Sciences*. The authors should be congratulated for investigating serial [^18^F]FDG PET/CT as a potential tool for characterizing immune-related metabolic changes in metastatic castration-resistant prostate cancer (mCRPC), an area of increasing relevance in the era of cancer immunotherapy [1].

Among the reported findings, the observation of increased splenic FDG uptake following treatment is particularly noteworthy. Such findings support the concept that FDG PET/CT may provide information not only regarding tumor metabolism but also regarding host immune activation. As immunotherapeutic strategies continue to expand, quantitative imaging biomarkers capable of reflecting treatment-induced immune responses are becoming increasingly important.

While the study focused on absolute splenic FDG uptake, we would like to highlight the potential value of normalized immune-organ biomarkers, particularly the spleen-to-liver ratio (SLR) and bone marrow-to-liver ratio (BLR). Absolute standardized uptake values (SUVs) can be influenced by several biological and technical factors, including blood glucose levels, body composition, scanner calibration, image reconstruction algorithms, and physiologic interindividual variability. Consequently, comparing absolute splenic uptake values across patients and studies may be challenging [2].

To mitigate these challenges, organ-to-liver ratios have been investigated as candidate measures of systemic immune and inflammatory activity. Practically, these metrics are typically obtained by drawing standardized volumes of interest (VOIs) to extract the mean SUV (SUVmean), using a reference region in the right lobe of the liver away from large vessels, and ensuring uniform scan timing (e.g., 50–70 minutes post-injection) across serial evaluations [2]. Growing evidence suggests that elevated SLR and BLR are associated with clinical outcomes in specific oncology settings. Wong et al. demonstrated that increased SLR was associated with clinical outcomes in metastatic melanoma patients treated with ipilimumab. Similarly, Zaba et al. reported that elevated BLR on pretreatment FDG PET/CT was independently associated with adverse survival outcomes in melanoma patients receiving anti-PD-1 therapy. More recently, Madsen et al. further supported the prognostic significance of PET-derived immune-organ biomarkers in patients treated with adjuvant immunotherapy [2,3,4].

However, it is critical to distinguish between evidence supporting the clinical and prognostic associations of these ratios and data demonstrating their technical reproducibility. While ratio-based biomarkers are hypothesized to facilitate quantitative standardization across institutions and imaging protocols in alignment with EANM recommendations, their superiority over absolute uptake measurements regarding robustness and technical reproducibility requires further formal validation. Furthermore, because these biomarkers utilize hepatic activity as the denominator, their validity depends inherently on the metabolic stability of the liver. Clinical situations in which hepatic metabolism is altered—such as diffuse hepatic metastases, non-alcoholic fatty liver disease (NAFLD), systemic therapy-induced hepatotoxicity, or active liver inflammation—could artificially skew the liver reference value. This would consequently alter the SLR or BLR independently of true background splenic or bone marrow activation, presenting a notable limitation to their widespread application [2].

Additionally, while data from melanoma cohorts provide a compelling rationale for exploring these metrics, these findings cannot be directly extrapolated to prostate cancer. Melanoma is a highly immunogenic, “hot” tumor characterized by profound, widespread T-cell infiltration and robust systemic lymphatic activation. Conversely, prostate cancer typically presents as an immunologically “cold” microenvironment with constrained systemic immune responsiveness and highly localized marrow-centric dynamics. SLR and BLR are not yet established predictive biomarkers of immunotherapy response, and their biological significance remains strictly unproven in mCRPC [2,3,4,5].

The observations reported by Panikar et al. provide an important foundation for future investigations of immune-response imaging in prostate cancer [1]. To translate these promising observations into clinical practice, future prospective trials in mCRPC should incorporate structured validation frameworks. Specifically, studies should rigorously evaluate:

- Longitudinal changes in SLR and BLR across pre- and post-treatment time points.
- Intra- and interobserver reproducibility of ratio measurements.
- Strictly harmonized PET acquisition and reconstruction algorithms to isolate biological changes from technical artifacts.
- Direct correlations with objective clinical outcomes (e.g., overall survival, radiographic progression-free survival) and circulating immune traits.
- The potential capability of these ratios to serve as early surrogate markers before conventional treatment response metrics manifest.

Importantly, our comments should not be interpreted as criticism of the study, which appropriately acknowledges its exploratory, hypothesis-generating nature. Rather, we view the authors’ work as an important step toward refining quantitative PET approaches for assessing treatment-induced immune activation. Rigorous, prospective clinical validation of these normalized immune-organ biomarkers within targeted prostate cancer immunotherapy trials is highly warranted to safely establish their ultimate utility in routine clinical care.

## Data Availability

No new data were created or analyzed in this study. Data sharing is not applicable to this article.
